# Supplementary material for: siRNA Nanoparticle Targeting PD-L1 Activates Tumor Immunity and Abrogates Pancreatic Cancer Growth in Humanized Preclinical Model
Source: Cells. 2021 Oct 13;10(10):2734. doi: 10.3390/cells10102734 (PMC8534711; doi:10.3390/cells10102734)
Supplement: Supplementary file 1 [file cells-10-02734-s001.zip › cells-1381872-supplementary.pdf]

Supplementary Material for

siRNA nanoparticle targeting PD-L1 activates tumor immunity and abrogates pancreatic cancer growth in humanized, orthotopic preclinical model

- This file contains 6 supplementary figures and 1 supplementary table.

Supplementary Figure 1

A

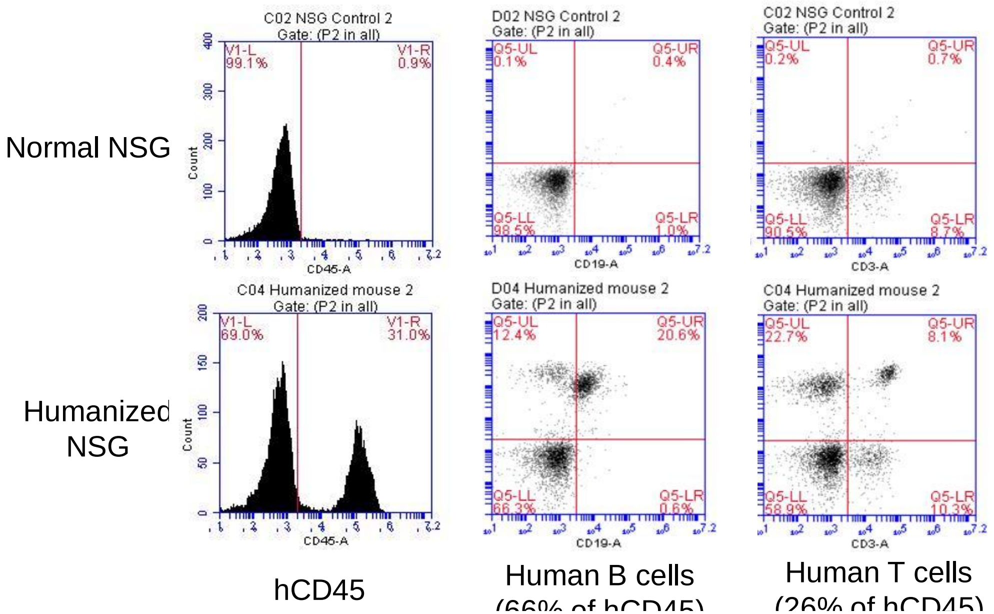

B

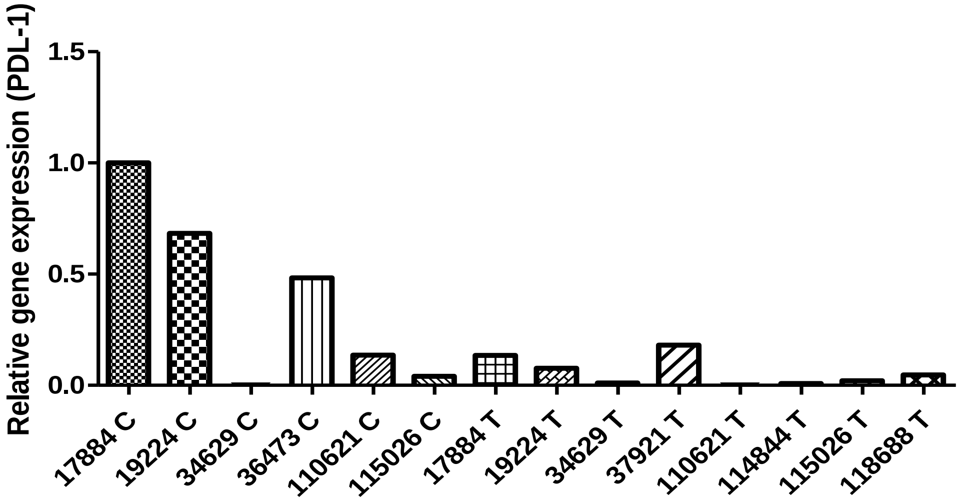

Figure S1. A. Representative flow cytometry plots showing human hematopoietic cells ($hCD45^+$), human T lymphocytes ($hCD45^+hCD3+$) and human B lymphocytes ($hCD45^+hCD19^+$) in the blood of humanized normal mice. B. Expression profile of PDAC primary cells (marked as C) and tumors (marked as T).

Supplementary Figure 2

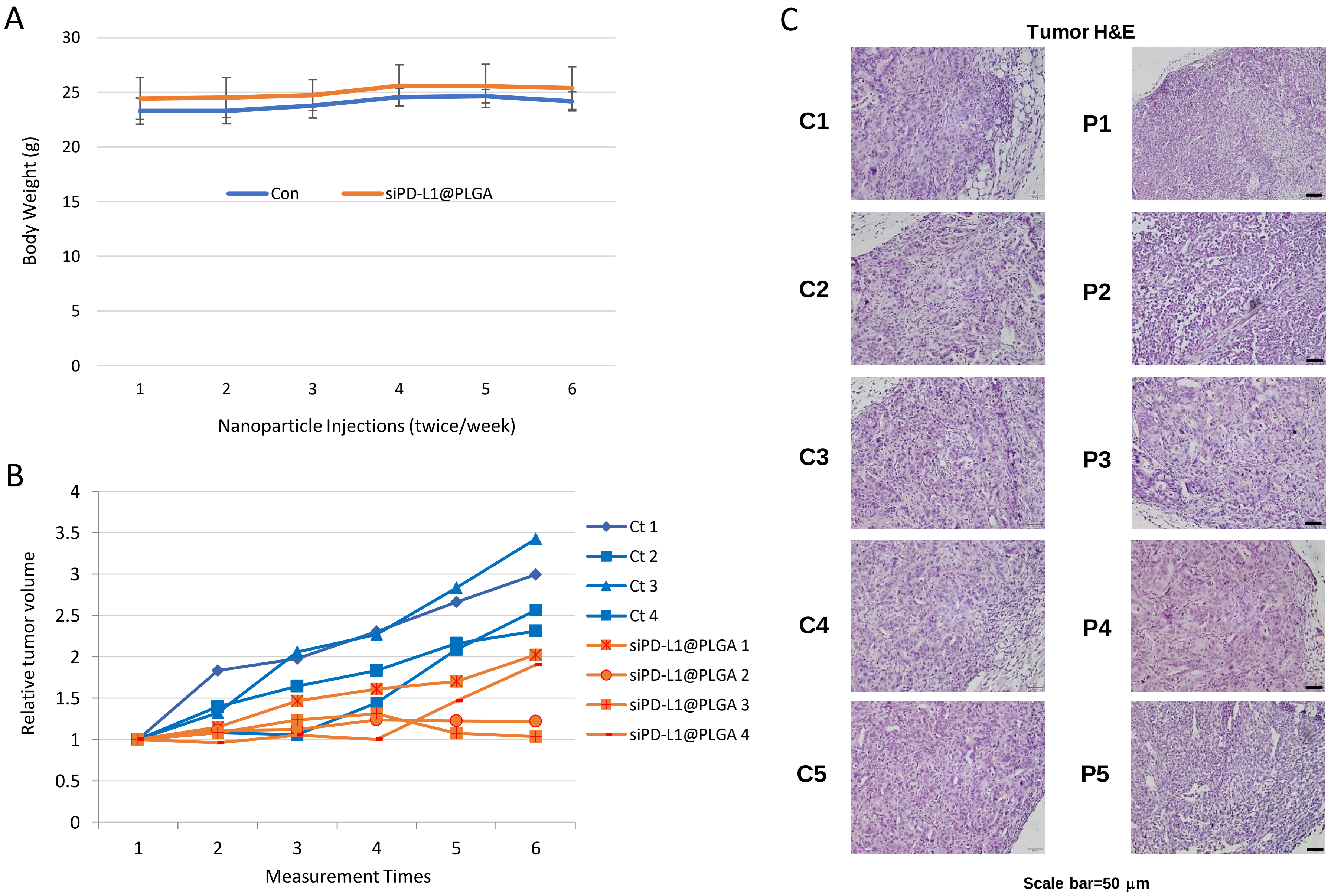

Figure S2. A. Graph for the body weight changes during the siPD-L1@PLGA treatment (in orange). B. Relative tumor volume of the individual mouse of control group (in blue) or siPD-L1@PLGA treated group (in orange). C. H&E staining of each tumor

Supplementary Figure 3

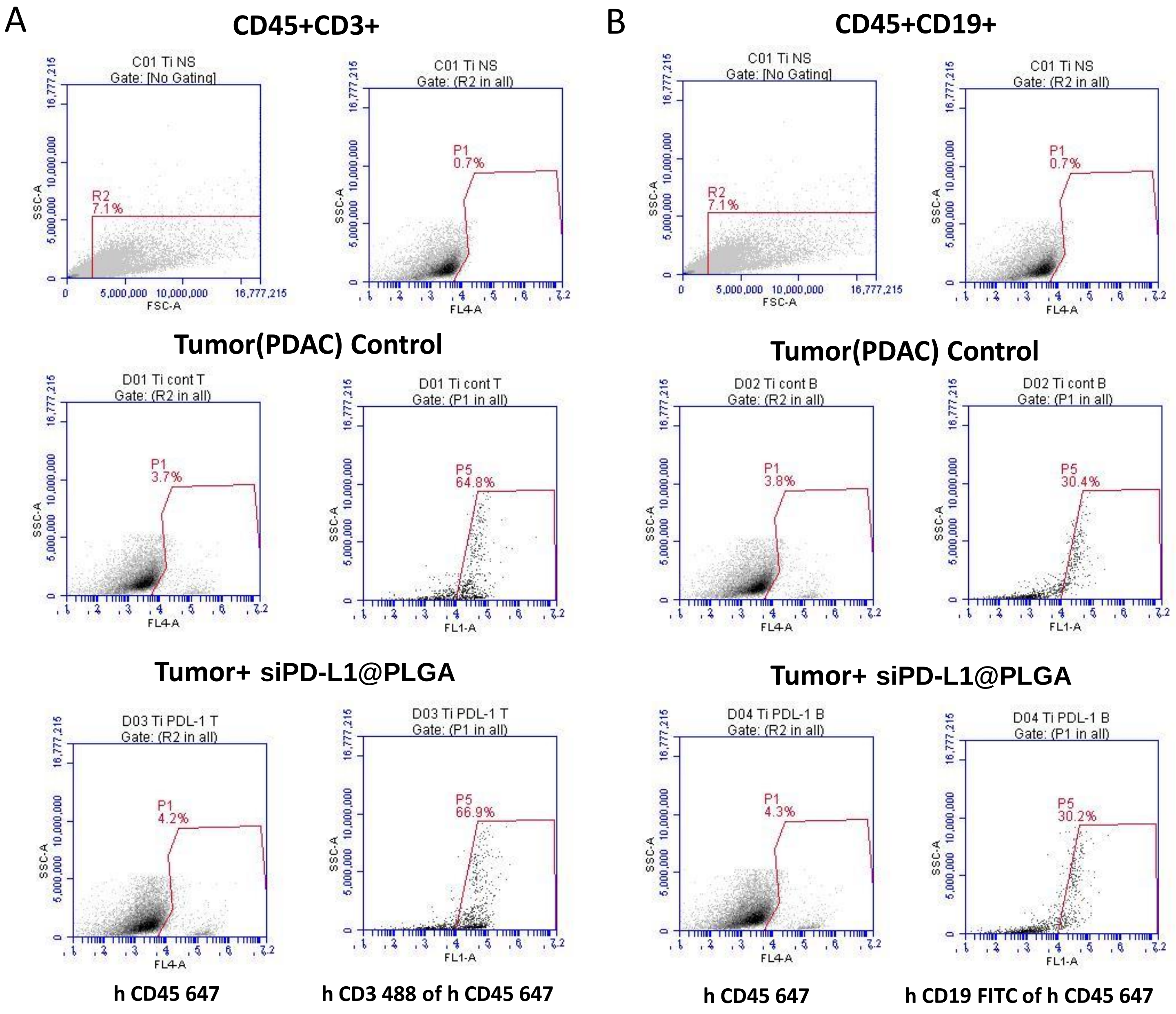

Figure S3. Representative flow cytometry plots and gatings for the tumor infiltrated immune cell analysis. Panels in A represent the gating for Cd45+Cd3+ cells, whereas panels in B represent Cd45+Cd19+ cells.

Supplementary Figure 4

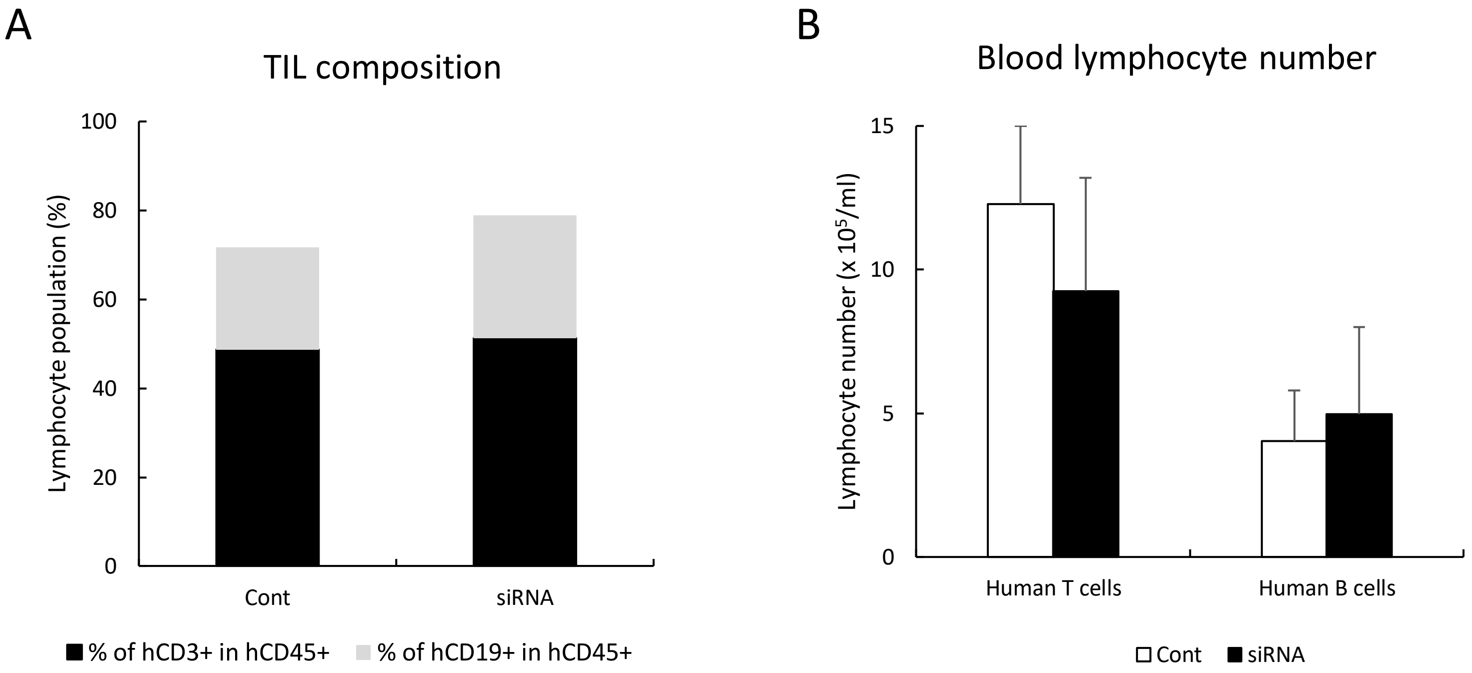

Figure S4. Human lymphocyte composition in tumor (A) and human lymphocyte count in the blood (B) of humanized NSG mice bearing PDAC tumors treated with vehicle or nano-PD-L1 siRNA. $hCD45^{+}hCD3^{+}$ and $hCD45^{+}hCD19^{+}$ represent human T lymphocytes and human B lymphocytes, respectively. Data are expressed as the mean ± SE (n=4-5 mice/group).

Supplementary Figure 5

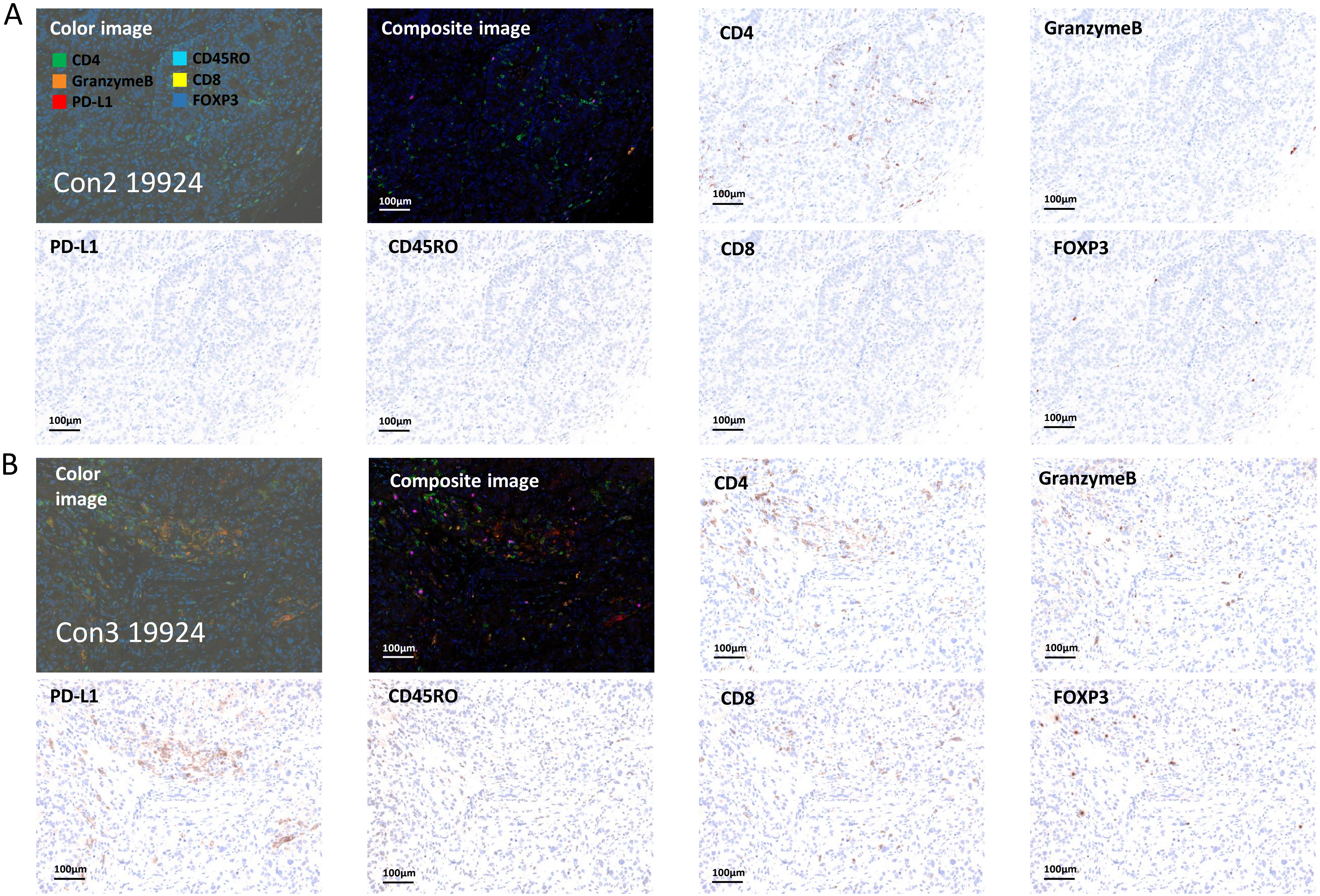

Figure S5. Raw data of OPAL images shown in Figure 5B and 5C. (A-D) present control tumors and (E-H) present siPD-L1@PLGA treated tumors

Supplementary Figure 5

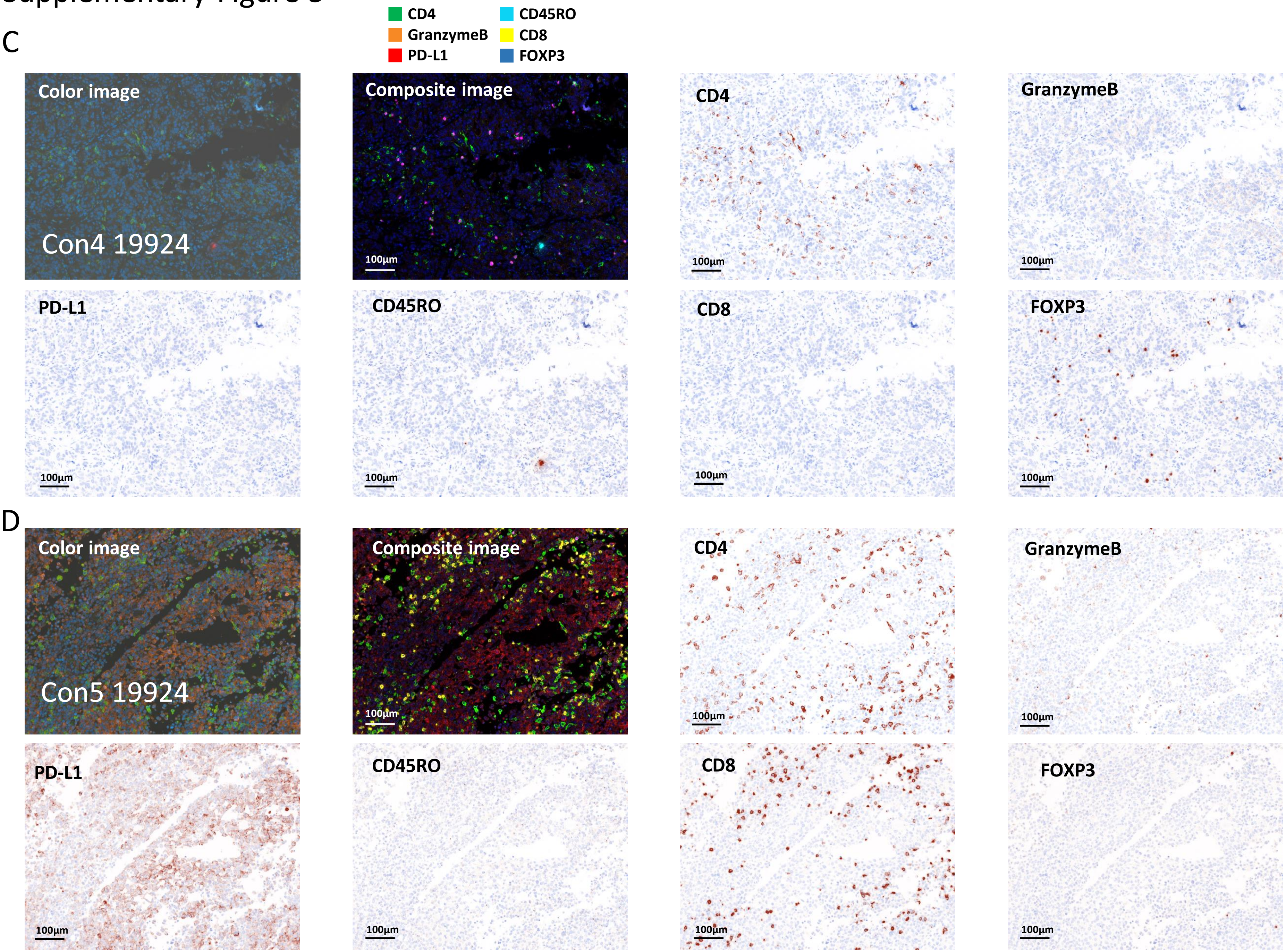

Supplementary Figure 5

E

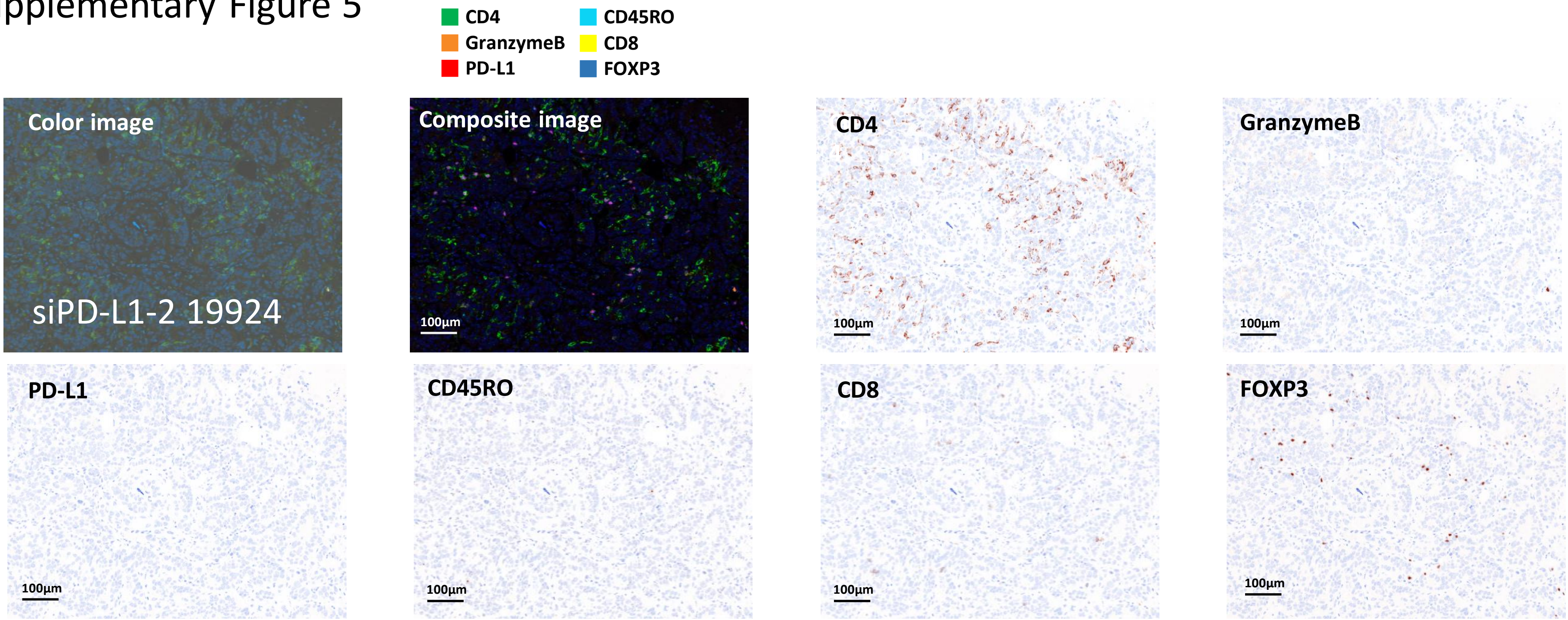

F

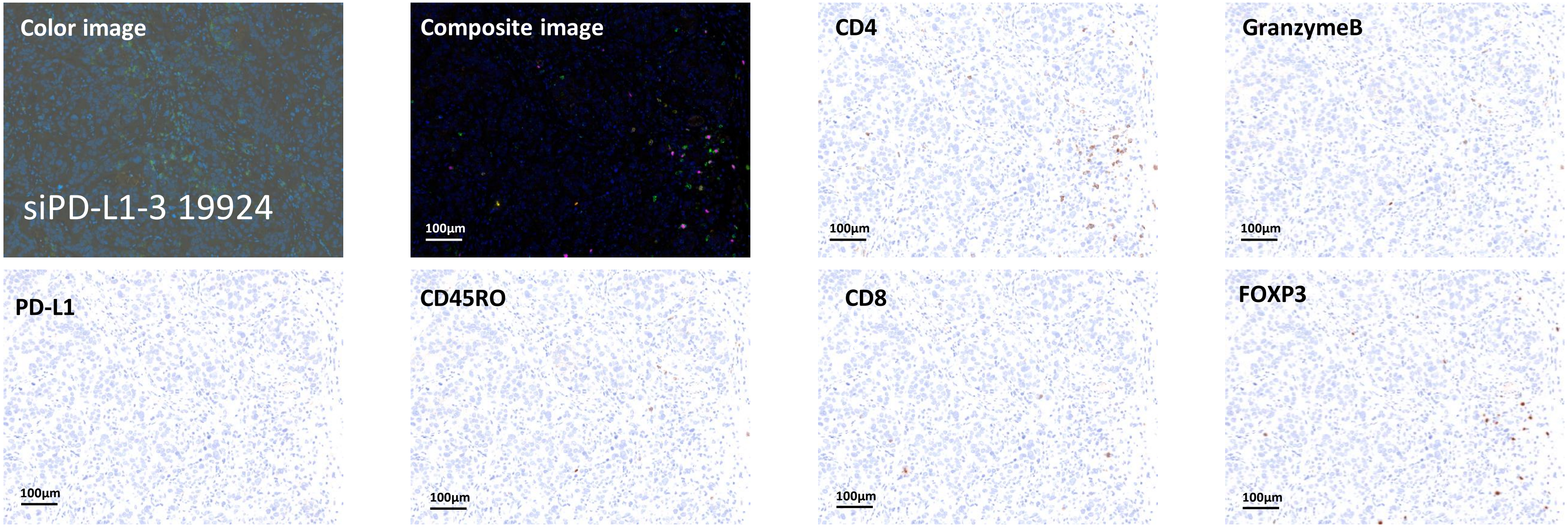

Supplementary Figure 5

G

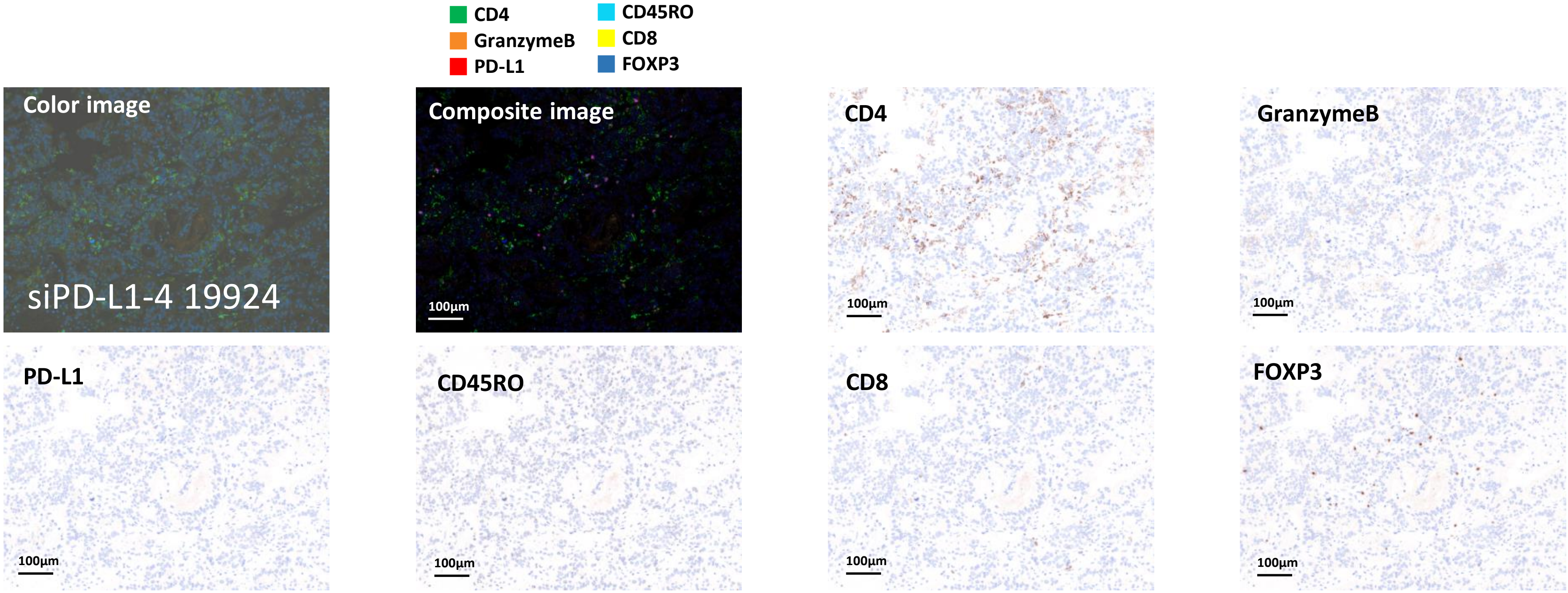

H

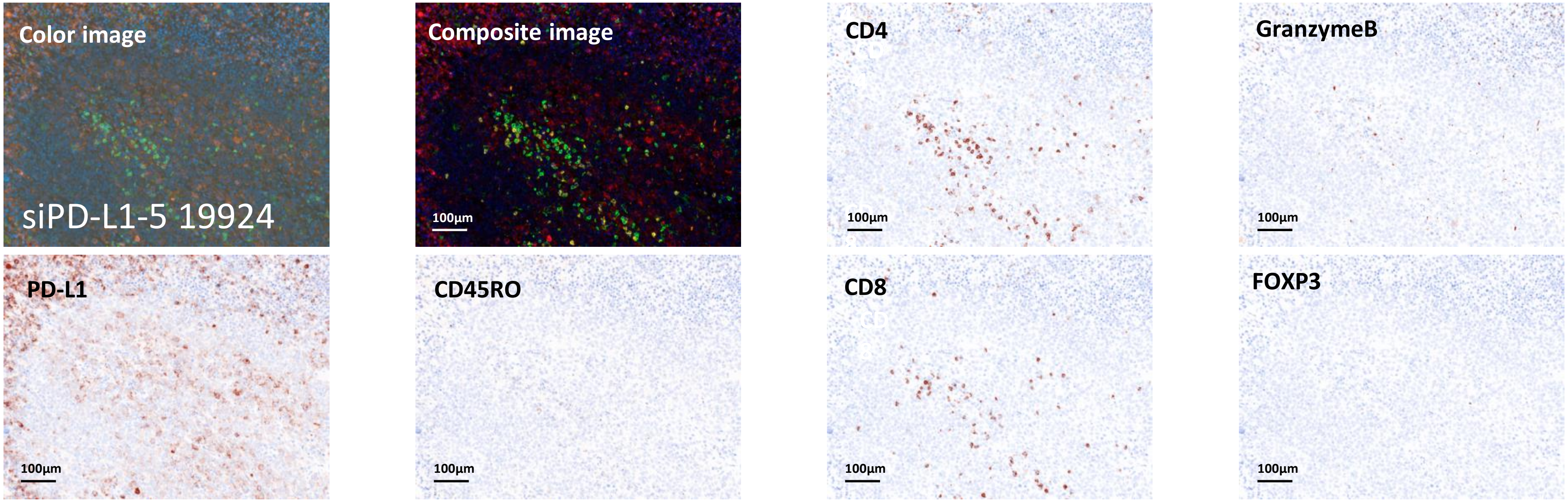

Supplementary Figure 6

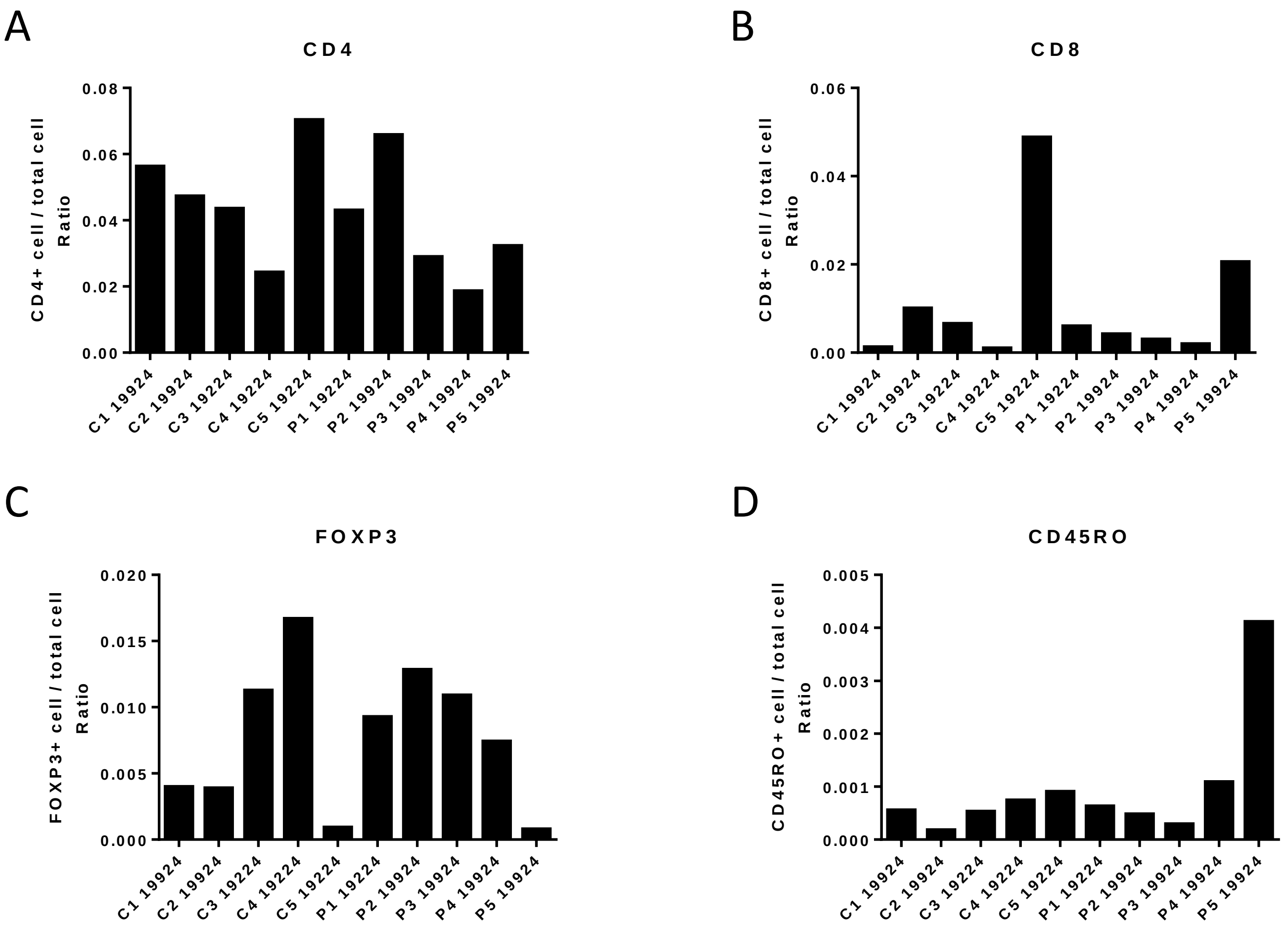

Figure S6. The ratio of infiltrated immune cells in the individual PDAC tumors. CD4(A), CD8(B), FoxP3(C) and CD45RO(D) were analyzed. C1-C5 indicate control tumors whereas P1-P5 indicate siPD-L1@PLGA treated tumors

Supplementary Table S1. Density of immune cells in the blood of normal NSG and humanized NSG mice. Representative flow cytometry analysis of the percentage of human hematopoietic cells (hCD45+), human T lymphocytes ($hCD45^+hCD3^+$) and human B lymphocytes ($hCD45^+hCD19^+$) in the blood of humanized normal mice

Mouse	Total cells (/200ul)	hCD45+	hCD45+ hCD19+	hCD45+ hCD3+
NSG 1	8.6×10^5	1.1 %	1.0 %	1.0 %
NSG 2	8.5×10^5	0.9 %	0.4 %	0.7 %
Hu NSG 1	3.8×10^5	27.1%	21.7 % (80 %)	4.6 % (17 %)
Hu NSG 2	3.9×10^5	30.8 %	20.6 % (66.9 %)	8.1 % (26.3 %)
